# Tumor necrosis factor superfamily 14 is critical for the development of renal fibrosis

**DOI:** 10.18632/aging.104151

**Published:** 2020-11-24

**Authors:** You Li, Ming Tang, Bo Han, Shun Wu, Shu-jing Li, Qian-hui He, Feng Xu, Gui-qing Li, Kun Zhang, Xu Cao, Quan-you Zheng, Jian Chen, Di Yang, Gui-lian Xu, Ke-qin Zhang

**Affiliations:** 1 Department of Nephrology, The First Affiliated Hospital of Army Medical University (Third Military Medical University), Shapingba 400038, Chongqing, China; 2 Department of Immunology, Basic Medicine College of Army Medical University (Third Military Medical University), Shapingba 400038, Chongqing, China; 3 Urinary Nephropathy Center, The Second Affiliated Hospital of Chongqing Medical University, Nanan 400065, Chongqing, China

**Keywords:** TNFSF14, Sphk1, renal fibrosis

## Abstract

Objective: Tumor necrosis factor superfamily protein 14 (TNFSF14) was recently identified as a risk factor in some fibrosis diseases. However, the role of TNFSF14 in renal fibrosis pathogenesis remains unknown.

Results: It was found that TNFSF14 levels were significantly increased both in UUO-induced renal fibrotic mice and in patients with fibrotic nephropathy, compared with those in controls. Accordingly, *Tnfsf14* deficiency led to a marked reduction in renal fibrosis lesions and inflammatory cytokines expression in the UUO mice. Furthermore, the levels of Sphk1, a critical molecule that causes fibrotic nephropathy, were remarkably reduced in *Tnfsf14* KO mice with UUO surgery. In vitro recombinant TNFSF14 administration markedly up-regulated the expression of Sphk1 of primary mouse renal tubular epithelial cells (mTECs).

Conclusion: TNFSF14 is a novel pro-fibrotic factor of renal fibrosis, for which TNFSF14 up-regulates Sphk1 expression, which may be the underlying mechanism of TNFSF14-mediated renal fibrosis.

Methods: We investigated the effect of TNFSF14 on renal fibrosis and the relationship between TNFSF14 and pro-fibrotic factor sphingosine kinase 1 (Sphk1) by using the unilateral urethral obstruction (UUO)-induced mice renal fibrosis as a model and the specimen of patients with fibrosis nephropathy, by Masson trichrome staining, immunohistochemistry, qRT-PCR, and western blot analysis.

## INTRODUCTION

The prevalence of chronic kidney disease (CKD) is high and increasing continuously [1, 2]. Renal fibrosis is a common outcome of a wide variety of CKD and is associated with compromised kidney functions, leading to eventual end-stage renal disease, for which the renal function needs to be ameliorated and/or mitigated by undergoing dialysis or kidney transplantation [3]. However, no effective therapy is available to inhibit or reverse renal fibrosis.

Renal fibrosis is characterized by tubular atrophy, inflammatory cell infiltration, activated myofibroblasts accumulation, and excessive extracellular matrix (ECM) deposition [4, 5]. Myofibroblasts, characterized by being α-smooth muscle actin (α-SMA) positive in renal interstitium, play an important role in the process of fibrosis and are proportionally correlated with severity of renal fibrosis [6]. In addition, many cytokines contribute to renal fibrosis, including TGF-β1, and directly promote ECM production in renal interstitium by epithelial cells and fibroblasts.

Tumor necrosis factor superfamily protein 14 (TNFSF14), also called as LIGHT or CD258, is a 29-kD type II transmembrane protein expressed primarily on activated T lymphocytes and other immunocytes [7]. TNFSF14 plays an important role in immune and inflammatory responses and can exist in a soluble form by proteolytic cleavage [8–10]. Recently, several studies have reported that TNFSF14 contributes to tissue remodeling and fibrosis, which are initiated by inflammatory conditions such as skin fibrosis, pulmonary fibrosis, and asthmatic airway remodeling, and rheumatoid arthritis [11–14]. In addition, TNFSF14 expression is induced by epithelial damage and directly increases the level of primary human bronchial epithelial cells (hBECs) undergoing EMT and expressing matrix metallopeptidase-9 [15]. However, the role of TNFSF14 in renal fibrosis pathogenesis remains unknown.

Sphingosine kinase 1 (Sphk1), an enzyme that produces sphingosine-1-phosphate (S1P), has gained considerable attention owing to its potential involvement in renal inflammation and fibrosis progression [16]. Sphk1 is up-regulated in inflammatory related kidney diseases, such as diabetic nephropathy and polycystic kidney disease [17, 18]. Moreover, it is well-established that Sphk1/S1P signaling promotes renal fibrosis by up-regulating miR-21, and Sphk1 silencing by siRNA treatment results in a reduction in fibronectin [19, 20]. However, how TNFSF14 pathway correlates Sphk1 expression during renal fibrosis progression is unclear.

In the present study, we have provided direct evidence, for the first time, that TNFSF14 is a novel pro-fibrotic factor in renal fibrosis progression, for which TNFSF14 up-regulates Sphk1 expression. Inhibition of the TNFSF14 pathway is plausible for the clinical treatment of patients with renal fibrosis.

## RESULTS

### Increased TNFSF14 expression in the fibrotic kidney

**Figure 1:**
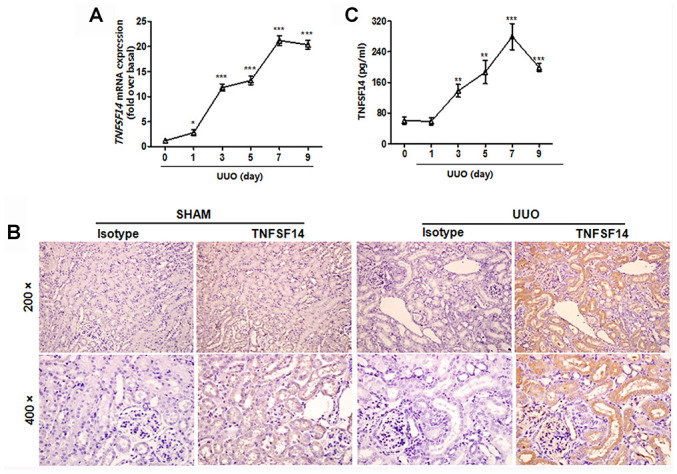
**Increased TNFSF14 expression in mice after UUO surgery.** (**A**) TNFSF14 expression in kidney tissues at the indicated time points in *Tnfsf14*^+/+^ mice after UUO surgery was assessed by qRT-PCR. GAPDH was used as the internal control. (**B**) The expression of TNFSF14 in the kidney tissues of *Tnfsf14*^+/+^ mice after UUO for 7 days was detected by immunohistochemistry (upper lane, original magnification ×200; lower lane, original magnification ×400). (**C**) TNFSF14 levels in serum in *Tnfsf14*^+/+^ mice at the indicated time points after UUO surgery were measured by ELISA. The data were representative of the results of three independent experiments. All values are represented as mean ± SEM. Sham group was used as the UUO control. n = 5 per group. ^*^P < 0.01, ^**^P < 0.01 and ^***^P < 0.001.

To address TNFSF14 relevance in determining kidney fibrosis, we used the unilateral ureteral obstruction (UUO)-induced renal fibrosis mouse model. It was found that TNFSF14 levels both in kidney tissues (Figure 1A and 1B) and in serum (Figure 1C) increased rapidly within 3 days and peaked at day 7 after UUO surgery (Figure 1A and 1C). TNFSF14 receptors, HVEM and LTβR, also increased remarkably in kidney tissues at day 7 after UUO surgery (Supplementary Figure 2A and 2B). Moreover, both TNFSF14 and HVEM/LTβR were primarily expressed in the tubular epithelia rather than in the interstitium (Figure 1B and Supplementary Figure 2).

**Figure 2:**
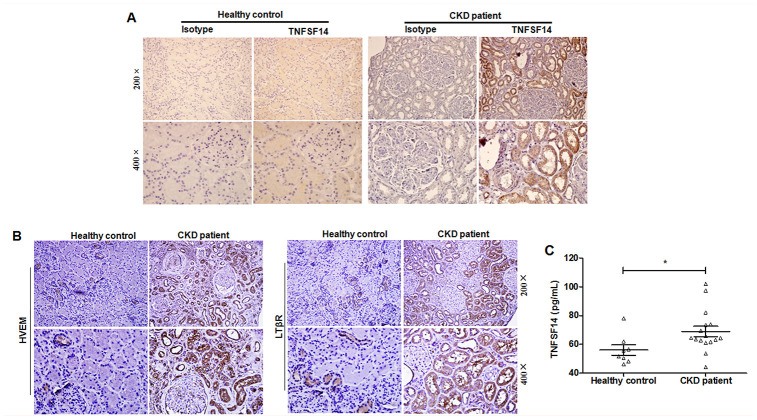
**Increased TNFSF14 expression in patients with fibrotic nephropathy.** (**A**) The expression of TNFSF14 in human kidney sections from healthy control, CKD patients were detected by immunohistochemistry (upper lane, original magnification×200; lower lane, original magnification×400). (**B**) The expression of TNFSF14 receptors (HVEM and LTβR) in biopsied human kidney specimens from patients with CKD were detected by immunohistochemistry. (upper lane, original magnification×200; lower lane, original magnification×400). Nontumoral kidney tissue from patients with renal cell carcinoma was used as healthy control. (**C**) TNFSF14 levels in serum from healthy controls (n = 8) and patients with CKD (n = 16) were measured by ELISA. The data were representative of the results of three independent experiments. Values are represented as mean ± SEM. ^*^P < 0.05.

Through specimen examination of patients with CKD (including membranous nephritis, focal segmental glomerulosclerosis and thrombotic microangiopathy), which showed fibrotic lesions in kidney tissues (Supplementary Figure 3), we also detected increased expression of TNFSF14 and its receptors in biopsy kidney tissues (Figure 2A and 2B) and in serum (Figure 2C).

These results suggest that TNFSF14 plays a critical role during renal fibrosis development.

### *Tnfsf14* deficiency reduces renal fibrosis

**Figure 3:**
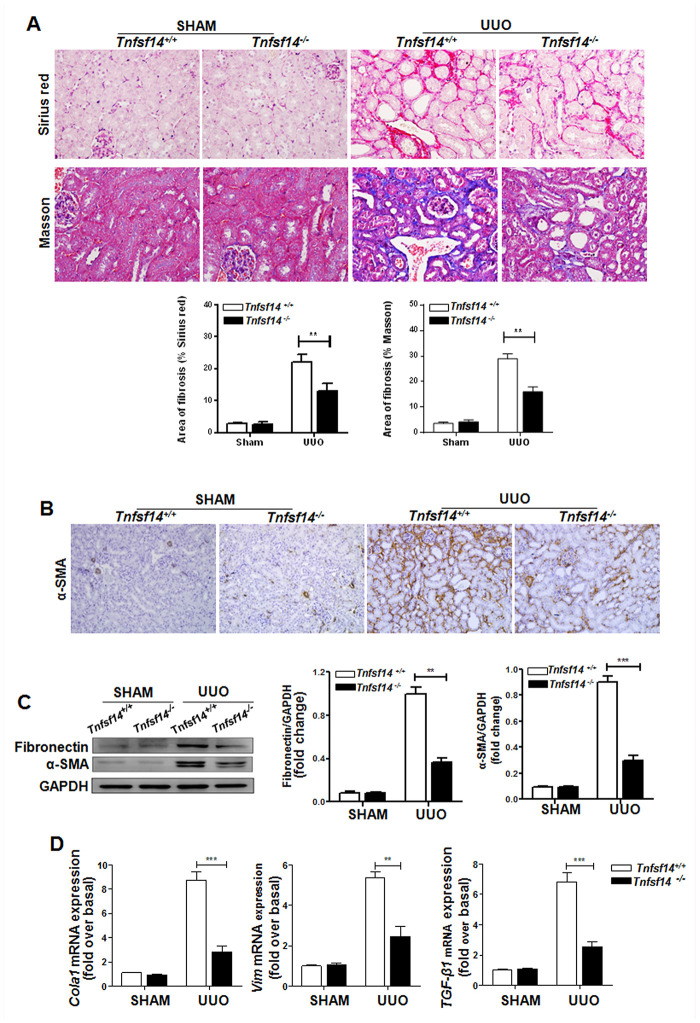
***Tnfsf14* deficiency ameliorates UUO-induced renal fibrosis. Kidney tissues from *Tnfsf14*^+/+^ and *Tnfsf14*^−/−^ mice were collected at 7 days after UUO surgery.** Sham group was used as the control of UUO. (**A**) Sirius Red and Masson staining of kidney tissues sections. Original magnification ×400. (**B**) α-SMA expression in kidney tissues was measured by immunohistochemistry. Original magnification ×200. (**C**) Western blot analyses of renal fibronectin and α-SMA expression in kidney tissues. Representative western blot (Left) and quantitative data (Right) are presented. (**D**) The *mRNA* levels of pro-fibrotic mediators *Cola1*, *Vim*, and *TGF-β1* were measured by qRT-PCR. The data were representative of the results of three independent experiments. All values are represented as mean ± SEM. n = 5 per group. ^**^P < 0.01 and ^*^^**^P < 0.001.

To further examine the functional importance of TNFSF14 in kidney fibrosis, we used *Tnfsf14*-deficient mice. *Tnfsf14* absence apparently alleviated UUO-induced tubular injury and renal fibrosis, as demonstrated by significant reductions in collagen deposition by staining with Sirius Red and Masson’s trichrome (Figure 3A), in the expression of α-SMA and fibronectin by immunohistochemical staining and western blot (Figure 3B and 3C), in the mRNA levels of pro-fibrotic markers *Cola1,* *Vim*, and *TGF-β1* by qRT-PCR (Figure 3D), and in tubular dilation, brush border disruption and tubular atrophy (Supplementary Figure 4A and 4B).

**Figure 4:**
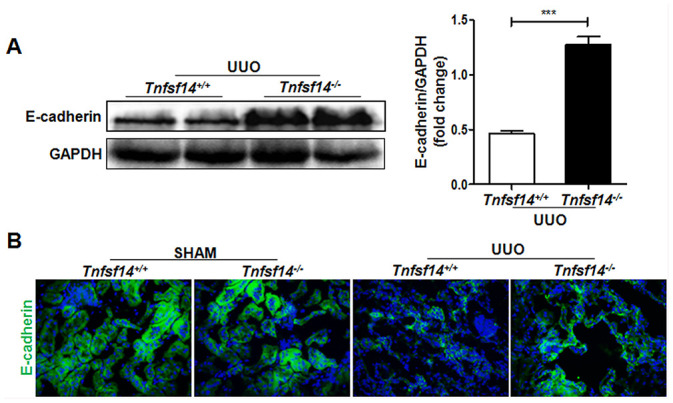
**Increased renal tubular cell integrity in *Tnfsf14*-deficient mice after UUO.** Kidney tissues of *Tnfsf14*^+/+^ and *Tnfsf14*^−/−^ mice were collected after UUO surgery for 7 days. (**A**) E-cadherin expression in kidney tissues was measured by western blot. Representative western blot (Left) and quantitative data (Right) are presented. (**B**) E-cadherin expression in kidney tissues was measured by immunofluorescence. Sham group was used as the control of UUO. The data were representative of the results of three independent experiments. Values are represented as mean ± SEM. Original magnification ×400. n = 5 per group. ^***^P < 0.001.

E-cadherin expression is an important marker of epithelial integrity and its absence is closely related to tissue fibrosis [21]. Therefore, we detected the expression of E-cadherin in the obstructed kidney, and found that it was significantly higher in *Tnfsf14* KO mice than that of WT mice after UUO surgery (Figure 4A and 4B).

### Tnfsf14 deficiency attenuates inflammatory cytokines expression in psoriatic skin lesions

**Figure 5:**
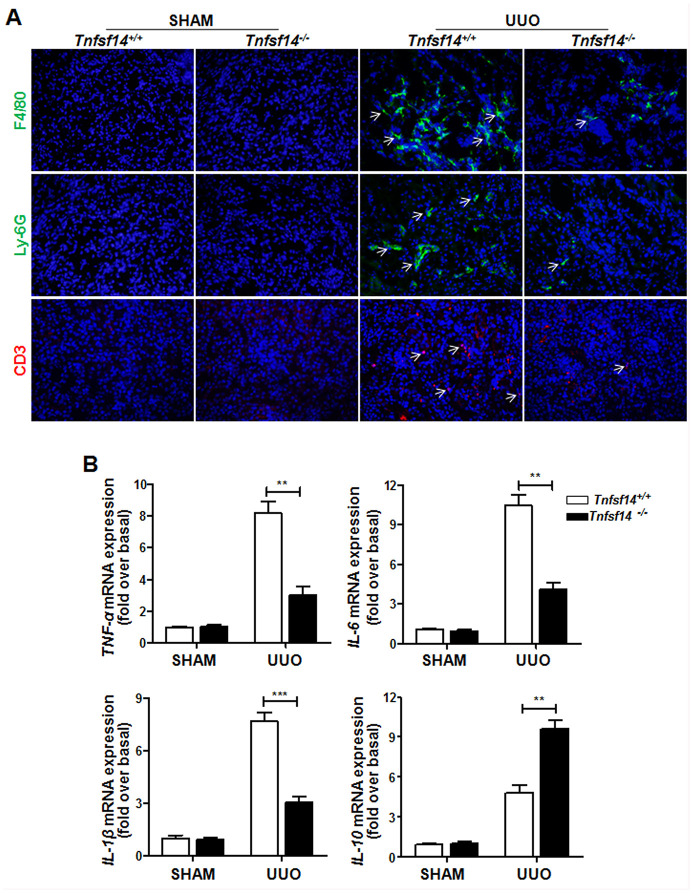
**Reduced inflammatory responses in *Tnfsf14*-deficient mice after UUO. Kidney tissues of *Tnfsf14*^+/+^ and *Tnfsf14*^−/−^ mice were collected after UUO surgery for 7 days.** (**A**) Infiltration of neutrophils (Ly-6G^+^, white arrows), macrophages (F4/80^+^, white arrows), and T lymphocytes (CD3^+^, white arrows) in kidney tissues was assessed by immunofluorescence. Original magnification ×400. (**B**) The mRNA levels of inflammatory cytokines *TNF-α*, *IL-6*, *IL-1β*, and *IL-10* in kidney tissues were measured by qRT-PCR. Sham group was used as the control of UUO. The data were representative of the results of three independent experiments. Values are represented as means ± SEM. n = 5 per group. ^**^P < 0.01 and ^***^P < 0.001.

Persistent inflammatory cell infiltration in the tissue and inflammatory cytokines secretion play an important role in renal fibrosis progression [22]. It was demonstrated that *Tnfsf14* deficiency led to a remarkable reduction in infiltration of macrophages (F4/80^+^), neutrophils (Ly-6G^+^), and T lymphocytes (CD3^+^) (Figure 5A) and in the mRNA levels of pro-inflammatory cytokines *TNF-α,* *IL-6, and IL-1β* (Figure 5B); However, an increase was observed in anti-inflammatory factor *IL-10* (Figure 5B) level in UUO-induced obstructed damaged kidney.

These data clearly demonstrate that *Tnfsf14* deficiency can alleviate disease severity during UUO-induced renal fibrosis in mice.

### Sphk1 is critical for the development of renal fibrosis

**Figure 6:**
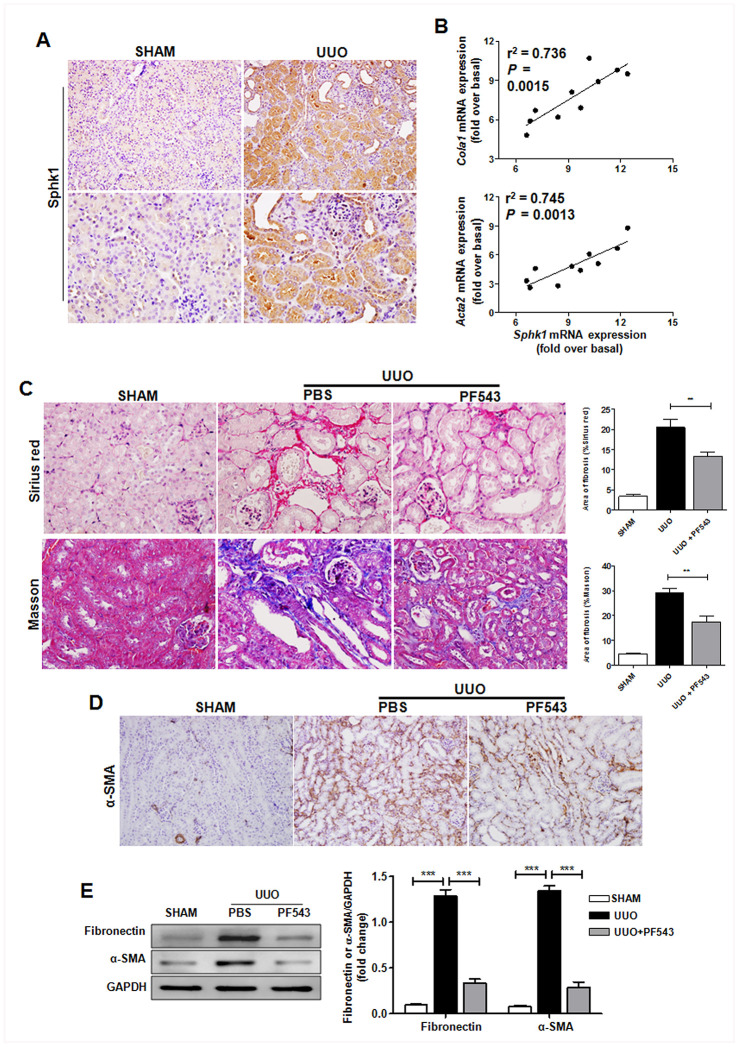
**Sphk1 is critical for UUO-induced kidney fibrosis in mice.** (**A**) Sphk1 expression in kidney tissues of *Tnfsf14*^+/+^ mice after UUO surgery for 7 days was measured by immunohistochemistry (upper lane, original magnification ×200; lower lane, original magnification ×400). (**B**) Linear regression showed a close correlation between *Sphk1* mRNA expression and *Cola1* and *Acta2* mRNA expression in kidney tissues of *Tnfsf14*^+/+^ mice after UUO surgery for 7 days. Spearman’s correlation coefficient and *P* value are shown (n = 10). (**C**–**E**) After UUO surgery, PF543 (1 mg/kg/day) was injected intraperitoneally for consecutive 7 days, and then kidney tissues were collected from each group. (**C**) Sirius Red and Masson staining of kidney tissues sections. Original magnification ×400. (**D**) α-SMA expression in kidney tissues was measured by immunohistochemistry. Original magnification ×200. (**E**) Western blot analyses of renal fibronectin and α-SMA protein in kidney tissues. Representative western blot (Left and quantitative data (Right) are presented. Sham group was used as the control of UUO. The data were representative of the results of three independent experiments. All values are represented as means ± SEM. n = 5 per group. ^***^P < 0.001.

Sphk1 participates in some types of tissue fibrosis, including pulmonary, liver, and cardiac fibrosis [23–26]. Moreover, Sphk1 is closely associated with CKD [27, 28] Consistent with these results and similar to increased Sphk1 expression in renal biopsy of patients with CKD (Supplementary Figure 5A), we found that Sphk1 was significantly up-regulated in the UUO-induced obstructed kidney in mice (Figure 6A and Supplementary Figure 5B). Furthermore, *Sphk1* expression exhibited a close association with fibrosis-related factors expression, including *Cola1* (Figure 6B, r^2^ = 0.736) and *Acta2* (Figure 6B, r^2^ = 0.745).

To further assess the role of Sphk1 during renal fibrosis development, we used PF543, a specific inhibitor of Sphk1 activation, to block Sphk1 endogenous activity *in vivo*, and found that PF543 treatment led to an evident down-regulation in UUO-induced collagen deposition (Figure 6C) and the expression of α-SMA (Figure 6D) and fibronectin (Figure 6E) in kidney tissues in mice. All these results suggest that Sphk1 is an important factor during UUO-induced renal fibrosis.

### TNFSF14 signaling is essential for pro-fibrotic factor Sphk1 production during the development of renal fibrosis

**Figure 7:**
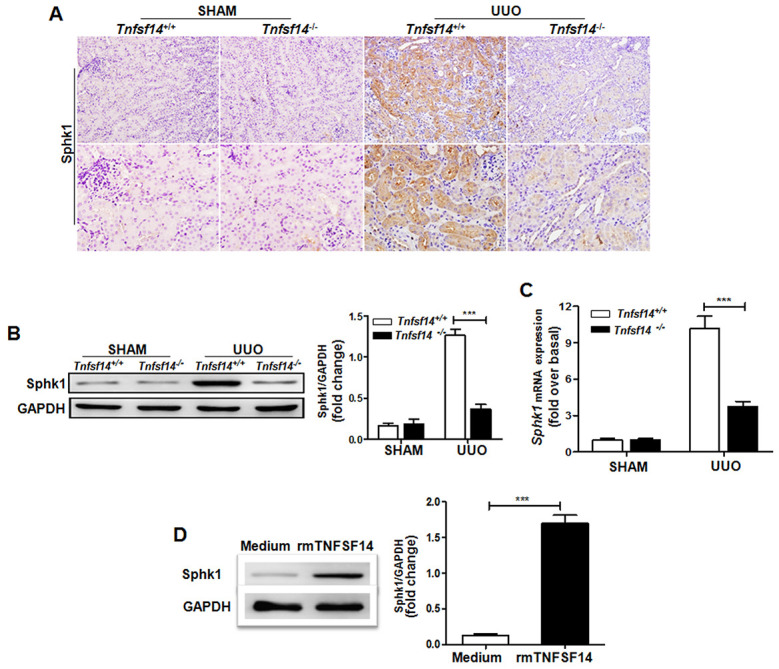
***Tnfsf14* deficiency leads to a remarkable reduction in UUO-induced Sphk1 expression in kidney tissues of mice.** After UUO surgery for 7 days, kidney tissues from *Tnfsf14*^+/+^ and *Tnfsf14*^−/−^ mice were collected. (**A**) The expression of Sphk1 in kidney tissues was measured by immunohistochemistry (upper lane, original magnification ×200; lower lane, original magnification ×400). (**B**) The expression of Sphk1 in kidney tissues was measured by western blot. Representative western blot (Left) and quantitative data (Right) are presented. (**C**) The expression of *Sphk1* mRNA in kidney tissues was measured by qRT-PCR. Sham group was used as the control of UUO. (**D**) Primary cultured mTECs were stimulated with rmTNFSF14 (100 ng/mL) for 24 h. Medium was used as the negative control. Western blot analyses of Sphk1 protein of mTECs. Representative western blot (Left) and quantitative data (Right) are presented. The data were representative of the results of three independent experiments. All values are represented as means ± SEM. n = 5 per group. ^**^P < 0.01 and ^***^P < 0.001.

To explore the association of pro-fibrotic factor Sphk1 with TNFSF14 pathway during renal fibrosis pathogenesis, we measured Sphk1 expression in *Tnfsf14*-deficient mice. Compared with *Tnfsf14*^+/+^ controls, *Tnfsf14*^−/−^ mice displayed significantly reduced Sphk1 expression in UUO-induced obstructed damaged kidney (Figure 7A–7C). To further investigate the effect of TNFSF14 signaling on Sphk1 expression, we isolated primary mTECs, which exhibited the typical characteristic cobblestone morphology of epithelial cells under light microscopy (Supplementary Figure 6A) and were CK-18, HVEM and LTβR positive (Supplementary Figure 6B). The Sphk1 production of primary cultured mTECs was remarkably increased after stimulating with rmTNFSF14 (100 ng/mL) for 24 h (Figure 7D and Supplementary Figure 7). Moreover, TNFSF14 showed a stronger ability for the induction of Sphk1 expression when compared with TNF-α, IFN-γ, TGFβ-1, IL-1β and IL-6 (Supplementary Figure 7). These data indicate that the TNFSF14 pathway is critical for pro-fibrotic factor Sphk1 expression during the development of renal fibrosis, which may be the underlying mechanism of TNFSF14-mediated renal fibrosis.

## DISCUSSION

TNFSF14 has been reported to be involved in various tissues fibrosis, including pulmonary and skin fibrosis [11, 12]. However, no studies had evaluated the role of TNFSF14 in renal fibrosis development. In the present study, we found that the expression of TNFSF14 and its receptors HVEM and LTβR were rapidly up-regulated in UUO-induced mouse renal fibrosis model and in patients with fibrosis nephropathy. Notably, although TNFSF14 is a transmembrane protein [7], it can be proteolyzed releasing a soluble, bioactive form since a metalloprotease cleavage site is present in TNFSF14 [29]. In agreement with previous results from the concanavalin A-induced hepatitis model [30], the morbidly obese subjects [31] and the diabetic patients [32], circulating TNFSF14 concentrations were significantly increased in fibrotic nephropathy patients. Furthermore, the important role of soluble form TNFSF14 is confirmed in the pathogenesis of liver inflammation [30] and DSS-induced colitis [8]. However, the cellular source of plasma levels of TNFSF14 is complex and not mentioned in the most of literatures since TNFSF14 is expressed and can also be shed from different types of immune-inflammatory cells, including T and B lymphocytes, monocytes/macrophages, granulocytes, spleen cells, and dendritic cells [33–35]. Although it was confirmed that platelets in the diabetic patients [32] or activated T cells in acute DSS-induced colitis [8] were the important cellular source of plasma levels of TNFSF14, the possibility could not be dismissed that other cell types might be capable of shedding TNFSF14. Herein, the source and the functions of increased circulating TNFSF14 in fibrotic nephropathy patients was not evaluated because of the limited human samples, and further studies are needed in the future.

“No inflammation, no fibrosis”, and in agreement with previous studies [2, 36], UUO induced a significant increase in the expression of several pro-inflammatory cytokines as well as pro-fibrotic factors. However, *Tnfsf14* deficiency markedly down-regulated UUO-induced inflammatory responses and renal fibrosis, which indicating that the protection observed in *Tnfsf14* deficient mice, at least partially, is associated to the reduced immune-inflammatory response. Consistent with these results, anti-inflammation drugs showed a potentially effectiveness for inflammatory renal diseases [37, 38].

There are several cell types that can generate proliferating and activated myofibroblast, such as resident fibroblasts, pericytes, epithelial-to-mesenchymal transition (EMT), endothelial-to-mesenchymal transition, and circulating bone-marrow-derived cells [39]. Here, our results showed a remarkable reduced α-SMA level in renal tissues of UUO-induced *Tnfsf14* KO mice, indicating a lower level of fibrosis and fewer myofibroblasts in kidney tissues. By using lineage tracing techniques, it was demonstrated that approximately 36% of fibroblasts in UUO-induced fibrosis were derived from tubular EMT [40]. TNFSF14 has been proved to induce EMT effectively [15]. That may be the reason why fewer myofibroblasts and then the decreased renal fibrosis were observed in UUO-induced *Tnfsf14* KO mice. On the other hand, several studies showed that epithelia contributed little to the myofibroblast population after injury [41–43]. The reasons for the discrepancy may be due to the different experimental models and animal strains [44]. In addition, because it is difficult to demonstrate EMT in vivo, and a concept of “partial EMT” is proposed in UUO model; partial EMT means that tubular epithelia acquire mesenchymal features but do not fully transform into myofibroblasts [45], which explains the conflicting views previously.

E-cadherin is essential for the maintenance of epithelial cell integrity and its absence is closely related to renal fibrosis [46, 47]. TNFSF14 directly induces E-cadherin degradation [15, 48]. Consistent with these results, our data showed that *Tnfsf14* deficiency substantially preserved E-cadherin expression. Taken together, these results indicate a profound involvement of endogenous TNFSF14 in kidney fibrosis pathogenesis.

Sphk1 plays a pivotal role in renal inflammatory response and fibrosis. Sphk1/S1P was shown to induce pro-fibrotic factors production by cross-activating the TGF-β/Smad signaling pathway [49], and over-expression of Shpk1 promoted the release of inflammatory factors leading to glomerulosclerosis and interstitial fibrosis [28]. *Sphk1* deficiency induced renal fibrosis to a lesser extent in diabetic mice [50]. In the present study, the pro-fibrotic properties of Sphk1 were further confirmed in the UUO-induced kidney fibrosis mouse model. In contrast to the pro-fibrotic role of Sphk1, it was found that *Sphk2-*deficient rather than *Sphk1*-deficient mice were protected from folic acid or ischemia-reperfusion injury-induced renal fibrosis [51]. The discrepancy between these results may be related to the different renal fibrosis models, and the pro-fibrotic effects of *Sphk1* were not obvious in acute kidney injury model. In addition, we found that UUO-induced Sphk1 expression was remarkably reduced in *Tnfsf14* KO mice, and *in vitro* recombinant TNFSF14 administration markedly up-regulated Sphk1 expression of mTECs, indicating TNFSF14 was required for Sphk1 production during renal fibrosis and TNFSF14 may mediate renal fibrosis by way of potentiating pro-fibrotic factor Sphk1 expression.

Collectively, our results provide direct evidence of the role of TNFSF14 pathway in kidney fibrosis development, indicating that disturbing the TNFSF14 signaling pathway can be a useful immunotherapeutic strategy for kidney fibrosis in humans. Moreover, this is the first report showing that the TNFSF14 pathway potentiates pro-fibrotic factor Sphk1 expression, which may be the underlying mechanism of TNFSF14-mediated renal fibrosis.

## MATERIALS AND METHODS

### Human serum and kidney biopsy samples

Human serum samples and kidney specimens were obtained from 16 patients with CKD stage 3 or 4 at the Southwest Hospital, Third Military Medical University, China. All patients were diagnosed with CKD for the first time. Serum samples were also obtained from healthy volunteers, considered as controls. After centrifugation at 3000 rpm for 10 min to remove debris, serum samples were aliquot and stored at −80° C for further study. Optimal cutting temperature compound (O.C.T, Sakura Finetek, USA)-embedded human kidney biopsy sections (4-μm) were prepared as described previously [52]. Peritumoral renal tissues from patients with renal cell carcinoma who underwent nephrectomy were used as normal controls. Informed consent was obtained from all patients included in this study, and all experiments were conducted according to the principles of the Declaration of Helsinki. The study was approved by the ethical committee of the First Affiliated Hospital (Southwest Hospital) of Third Military Medical University.

### Animal models

Male C57BL/6 mice were purchased from the Peking University Animal Center (Beijing, China). *Tnfsf14*^−/−^ mice with a C57BL/6 genetic background were provided by Prof. Pfeffer (Institute of Medical Microbiology and Hospital Hygiene, University of Duesseldorf, Germany). Genotyping for *Tnfsf14* in mice was performed by PCR with the following primers, provided by Dr. Preffer [53]. For *Tnfsf14*, wild type: 5’-CGACAGACATGCCAGGAATGG-3’; common: 5’-ACG CATGTGTCCTGCGTGTGG-3’; mutant: 5’-GACGTAAACTCCTCTTCAGAC-3. Eight to twelve-week-old male mice were used for the present study. UUO-induced renal fibrosis was performed as described previously [54]. Briefly, mice were anesthetized with 1 % pentobarbital (10 μL/g), and the left ureter was exposed by a midline incision. The ureter was obstructed by two point ligations with 6–0 silk sutures. Sham-operated mice had their ureters exposed and manipulated and underwent the same procedure but were not ligated. The incision was sutured, and mice were allowed to recover and were provided *ad libitum* access to food and water. Mice were euthanized at day 1, 3, 5, 7, or 9 after the surgery, and kidney tissues and serum were collected for further study. For assessing PF543 therapeutic efficacy (Sphk1 activation specific inhibitor) on UUO-induced kidney fibrosis, three groups of mice were used: (1) Sham control, (2) UUO injected with vehicle, and (3) UUO injected with PF543. There were 5 mice in each group except for special instructions. Mice were treated with vehicle (PBS) or PF543 (1 mg/kg, Medchemexpress, USA) by intraperitoneal injection daily and were euthanized at 7 days after UUO. This dosage of PF543 is widely used in studies including in UUO-induced mouse model of renal fibrosis [55, 56], and the inhibitory effect of PF-543 was also determined by western blot (Supplementary Figure 1). All animal studies were approved by the Institutional Animal Care and Use Committee of the Third Military Medical University.

### Primary mouse renal tubular epithelial cells (mTECs) culture and treatment

mTECs were isolated as previous described [57, 58]. Briefly, the kidney was removed and washed with cold PBS. After the capsule was removed, kidney cortices from WT mice were cut into pieces and digested with collagenase (2 mg/mL) at 37° C for 30 min, followed by PBS washing. Next, the suspension was passed through a series of cell sieves (mesh diameters of 100 and 70 μm). Cortical tubular cells were centrifuged at 1300 rpm for 5 min, followed by PBS washing. Cells were cultured in DMEM/F12 medium supplemented with 10% fetal bovine serum (ScienCell, Carlsbad, USA) and 100 U/mL penicillin/streptomycin (Life Technologies, Grand Island, USA). For *in vitro* studies, serum-starved mTECs were stimulated with recombinant murine TNFSF14 (100 ng/mL), TNF-α (10 ng/mL), IFN-γ (100 ng/mL), TGFβ-1 (10 ng/mL), IL1-β (10 ng/mL), or IL-6 (10 ng/ml) (All from Peprotech, Rocky Hill, NJ, USA) for 24 h.

### Immunofluorescence staining

Kidney cryosections were fixed with 4 % paraformalin for 15 min at room temperature. mTECs cultured on coverslips were fixed with cold acetone for 10 min at −20° C. After blocking with 5 % BSA for 1 h, the slides were immunostained with primary antibodies against fibronectin, E-cadherin, CD3, CK-18, LTβR (All diluted by 1:100, Abcam, Cambridge, MA, USA), and TNFSF14, F4/80, Ly-6G, HVEM (All diluted by 1:100; Santa Cruz, Dallas, TX, USA). These slides were then stained with DyLight- or Cy3-conjugated secondary antibody (1:300; Biolegend, San Diego, CA, USA), respectively. Nuclei were stained using Hoechst33258 (Enzo, Lausen, Switzerland). The slides were visualized by fluorescent microscopy (Olympus BX51, Japan).

### Histology and immunohistochemistry

Kidney tissues were fixed in 4 % formalin and embedded in paraffin. Paraffin sections (4-μm) were stained with Masson’s trichrome and Sirius Red. Renal fibrosis was assessed and quantified by imaging analysis (ImageJ software; Bethesda, MD, USA). Briefly, 6–8 corticomedullary junction viewing fields were selected from appropriate areas for each kidney examined. Fibrosis was expressed as the percentage of the total area. A total of 4–5 images were evaluated per kidney, and mean values were calculated. Immunohistochemical staining was performed on kidney by using routine protocols [59]. Briefly, sections were blocked with 5 % BSA for 1 h at room temperature and incubated at 4° C overnight with primary antibodies against α-SMA, LTβR, Sphk1 (All diluted by 1:150; Abcam, Cambridge, MA, USA), and TNFSF14, HVEM (All diluted by 1:150; Santa Cruz, Dallas, TX, USA). These slides were then stained with a horseradish peroxidase-conjugated secondary antibody (1:800; Beyotime, Shanghai, China). The results were analyzed using the DAB assay kit (ZSGB-BIO, Beijing, China). The slides were visualized by microscopy (Olympus BX51, Japan).

### Quantitative real-time PCR

Total RNA was extracted from the tissues using the TRIzol reagent (Takara, Tokyo, Japan) according to the manufacturer’s protocol. First-strand cDNA was synthesized using a reverse transcription system (Takara, Tokyo, Japan) according to the manufacturer’s instruction, and the cDNA was used for quantitative real-time PCR analysis using SYBR Premix Ex Taq (Takara, Tokyo, Japan). mRNA levels were normalized to those of GAPDH. Primer sequences used for amplifications are presented in Table 1. All samples were measured in triplicates. Differences in gene expression were calculated using 2^−ΔΔct^ method.

**Table 1:** Sequences of primers used for qRT-PCR.

**Gene**	**Forward primer**	**Reverse primer**
Tnfsf14	ATCTTACAGGAGCCAACGCC	ACGTCAAGCCCCTCAAGAAG
Acta2	CCCAGACATCAGGGAGTAATGG	TCTATCGGATACTTCAGCGTCA
Cola1	TTCTCCTGGCAAAGACGGAC	CTCAAGGTCACGGTCACGAA
Vim	CAAACGAGTACCGGAGACAG	TAGCAGCTTCAAGGGCAAAA
TGF-β1	CTCCCGTGGCTTCTAGTGC	GCCTTAGTTTGGACAGGATCTG
TNF-α	CCTGTAGCCCACGTCGTAG	GGGAGTAGACAAGGTACAACCC
IL-1β	GCAACTGTTCCTGAACTCAACT	ATCTTTTGGGGTCCGTCAACT
IL-6	TTCCTCTGGTCTTCTGGAGT	GTGACTCCAGCTTATCTCTTGG
IL-10	GCTGGACAACATACTGCTAACC	ATTTCCGATAAGGCTTGGCAA
Sphk1	TTTGGAGGTTGCTGACGAG	GGGGCGGCCAGATTTTTAG
GAPDH	GGTTGTCTCCTGCGACTTCA	TAGGGCCTCTCTTGCTCAGT

### ELISA

TNFSF14 levels in serum were measured using commercially available ELISA kit (Cloud-Clone, Houston, USA) according to the manufacturer’s instructions. OD values were detected using a microplate absorbance reader (BIO-RAD, California, USA) at a wavelength of 450 nm and calculated in the linear part of the curve.

### Western blot

Total proteins were isolated from the renal tissues using the RIPA buffer, and protein concentrations were quantified using the BCA protein assay kit (Beyotime, Shanghai, China). Protein samples (35 μg/lane) were resolved via sodium dodecyl sulfate-polyacrylamide gel electrophoresis and were transferred onto polyvinylidene difluoride membranes (Beyotime, Shanghai, China). The membranes were incubated overnight at 4° C with rabbit anti-mouse fibronectin (1:1000), mouse anti-mouse α-SMA (1:200), mouse anti-mouse E-cadherin (1:500), rabbit anti-mouse Sphk1 (1:1000) (All from Abcam, Cambridge, MA, USA) followed by incubation with horseradish peroxidase-conjugated goat anti-mouse or goat anti-rabbit IgG secondary antibodies (1:3000; ZSGB-BIO, Beijing, China). Immunoblots were visualized using the ECL Western blot Detection System (Millipore, Billerica, MA, USA). GAPDH was used as the loading control.

### Statistics

All data are represented as mean ± SEM. Statistical significances between experimental and control groups were assessed by the Student’s *t*-test or one-way ANOVA. Spearman (nonparametric) correlation analysis was used to assess the relationship between *Sphk1* mRNA expression in kidney and other variables. *P* < 0.05 was considered significant.

## Supplementary Material

Supplementary Figures
