# Supplementary material for: Tumor necrosis factor superfamily 14 is critical for the development of renal fibrosis
Source: Aging (Albany NY). 2020 Nov 24;12(24):25469–86. doi: 10.18632/aging.104151 (PMC7803499; doi:10.18632/aging.104151)
Supplement: Supplementary Figures [file aging-12-104151-s001.pdf]

SUPPLEMENTARY FIGURES

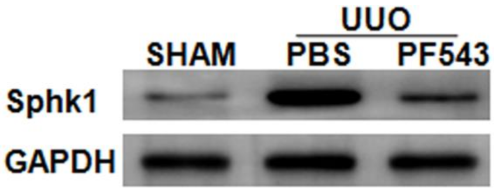

Supplementary Figure 1. Inhibition efficiency of PF543 treatment. After UUO surgery, mice were treated with PBS or PF543 (1 mg/kg) by intraperitoneal injection daily. Mice were euthanized and kidney tissues were collected at 7 days after UUO. Sham group was used as the control of UUO. The expression of Sphk1 was measured by western blot. GAPDH was used as a loading control. The data were representative of the results of three independent experiments.

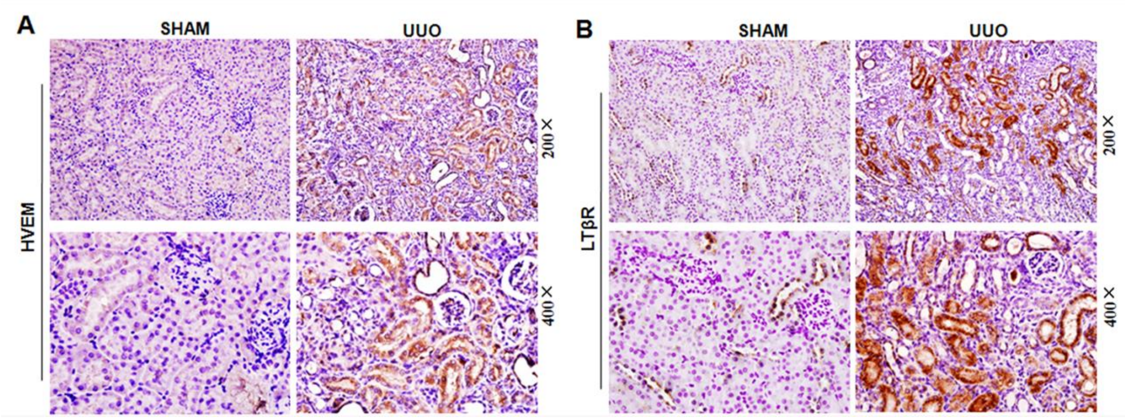

Supplementary Figure 2. Immunostaining of kidney tissues of mice with UUO surgery. The expression of HVEM (**A**) and LTβR (**B**) in kidney tissues on day 7 after UUO surgery was measured by immunohistochemistry. Sham group was used as the control of UUO. Upper lane, original magnification ×200; lower lane, original magnification ×400.

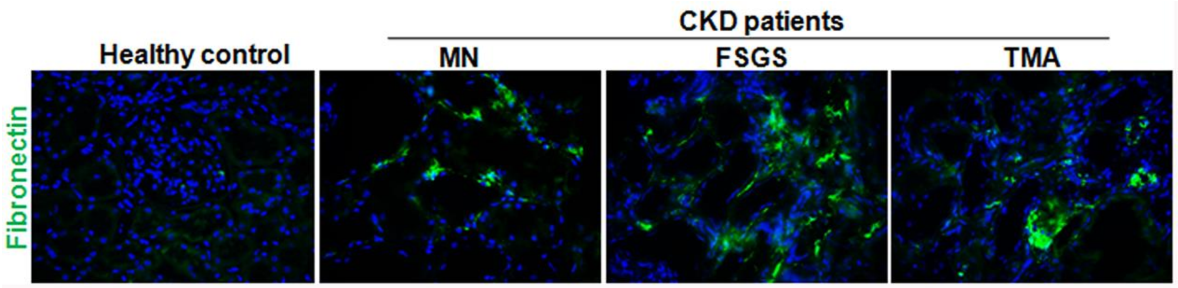

Supplementary Figure 3. Immunostaining of kidney tissues of CKD patients. Immunofluorescence staining of fibronectin in human kidney sections from healthy control, membranous nephritis (MN), focal segmental glomerulosclerosis (FSGS) and thrombotic microangiopathy (TMA). Original magnification ×400. The data were representative of the results of three independent experiments.

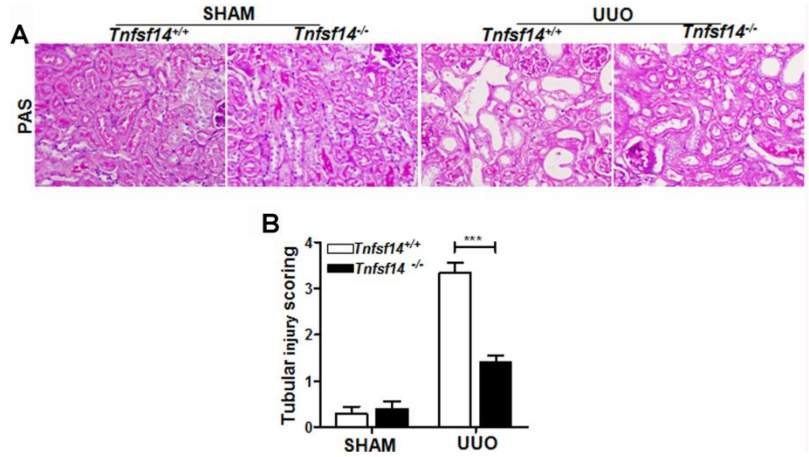

Supplementary Figure 4. Histopathology of kidney tissues from *Tnfsf14*$^{+/+}$ and *Tnfsf14*$^{-/-}$ mice. Kidney tissues were collected on day 7 after UUO surgery. Sham group was used as the control of UUO. (**A**) Kidney tubular injury was determined by using PAS staining. (**B**) Tubular injury scoring of kidney tissues. Original magnification ×400. The data were representative of the results of three independent experiments. All values are represented as mean ± SEM. n = 5 per group. $^{***}P < 0.001$.

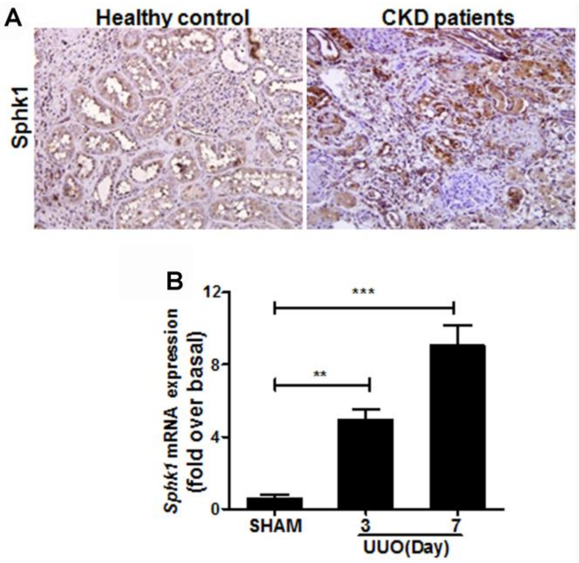

Supplementary Figure 5. Increased Sphk1 expression in fibrotic kidney. (**A**) Expression of Sphk1 in kidney sections from healthy control and patients with CKD was assessed by immunohistochemical staining. Original magnification ×200. (**B**) Expression of *Sphk1* in kidney tissues of *Tnfsf14*$^{+/+}$ mice at 3 and 7 days after UUO surgery was assessed by qRT-PCR. Sham group was used as the control of UUO. The data were representative of the results of two independent experiments. Values are represented as mean ±SEM. n = 5 per group. $^{**}P < 0.01$ and $^{***}P < 0.001$.

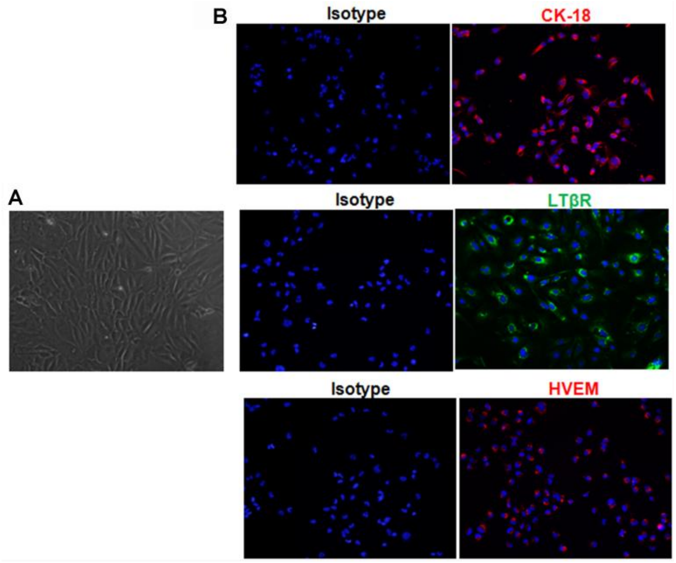

Supplementary Figure 6. Characteristics of primary mouse renal tubular epithelial cells (mTECs). (**A**) The typical characteristic cobblestone morphology of mTECs. (**B**) The expression of CK-18, LTβR, and HVEM in mTECs was assessed by immunofluorescence. The isotype here was used as the negative control of the corresponding primary antibody staining group during the immunofluorescence staining. The section in isotype group was treated with the corresponding second antibody, but not treated with the primary antibody. Nuclei were stained with Hoechst33258. Original magnification ×200. The data were representative of the results of three independent experiments.

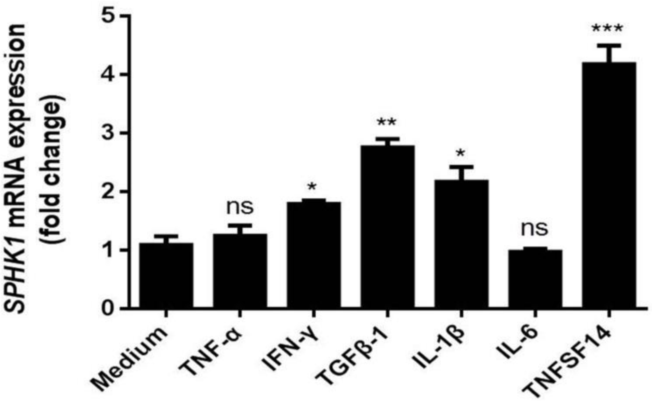

Supplementary Figure 7. Effect of inflammatory cytokines on Sphk1 production by renal tubular epithelial cells (mTECs). The expression of Sphk1 mRNA in mTECs was measured by qRT-PCR. Primary cultured mTECs were stimulated with recombinant murine TNFSF14 (100 ng/mL) , TNF-α (10 ng/mL), IFN-γ (100 ng/mL), TGFβ-1 (10 ng/mL), IL1-β (10 ng/mL), or IL-6 (10 ng/mL) for 24 h. Medium was used as the negative control. GAPDH was used as the internal control. The data were representative of the results of three independent experiments. All values are represented as means ± SEM. n = 5 per group. $^{*}P < 0.05$, $^{**}P < 0.01$ and $^{***}P < 0.001$.
